# Association between single nucleotide polymorphisms in vitamin D related and parathyroid hormone genes with nonsyndromic oral cleft

**DOI:** 10.1007/s00056-025-00614-w

**Published:** 2025-09-29

**Authors:** Jana Marciniak, Gabriela Fonseca-Souza, Rafaela Scariot, Svenja Beisel-Memmert, Nikolaos Daratsianos, Michelle Nascimento Meger, Thais Vilalba Paniagua Machado do Nascimento, Christian Kirschneck, Juliana Feltrin-Souza, Erika Calvano Küchler

**Affiliations:** 1 Department of Orthodontics, University Hospital Bonn, Medical Faculty, Welschnonnenstr. 17, 53111 Bonn, Germany; 2 Department of Stomatology, Federal University of Paraná, Curitiba, Paraná, Brazil; 3 University of Tuiuti of Paraná, Curitiba, Paraná, Brazil

**Keywords:** Cleft palate, Genetic polymorphism, Congenital craniofacial abnormalities, Parathyroid hormone, Real-time polymerase chain reaction, Gaumenspalte, Genetischer Polymorphismus, Kongenitale kraniofaziale Anomalien, Parathormon, Echtzeit-Polymerase-Kettenreaktion

## Abstract

**Objective:**

Nonsyndromic oral clefts (NSOC) result from disruptions in the development of the primary and secondary palate and are categorized into cleft lip (CL), cleft lip and palate (CLP) and cleft palate (CP). NSOC is a condition with complex genetically heterogeneous backgrounds for that several genes might be involved. Thus, this study investigated whether functional single nucleotide polymorphisms (SNPs) in genes that potentially influence the vitamin D levels and parathyroid hormone (PTH) levels are associated with NSOC.

**Materials and methods:**

Brazilian individuals with NSOC—presenting cleft lip (CL), cleft lip and palate (CLP) and cleft palate (CP)—and a comparison group were selected. Individuals’ DNA was obtained from epithelial cells of the oral mucosa. Nine SNPs in five genes (*VDR, CYP27B1, CYP24A1, SEC23A* and *PTH*) were selected. Genotyping was performed by real-time polymerase chain reaction (PCR). The association between NSOC and vitamin D related polymorphisms was analyzed by Χ^2^ test at a significance level of *p* < 0.005 (after Bonferroni correction).

**Results:**

The sample consisted of 274 individuals: 154 NSOC individuals and 120 controls. A total of 43 (27.9%) had CL, 91 (59.1%) had CLP and 20 (13.0%) had CP. After Bonferroni correction, none of the SNPs were associated with NSOC (*p* > 0.005).

**Conclusion:**

None of the studied SNPs in vitamin D related and parathyroid hormone genes were associated with NSOC.

## Abstract (de)

**Zielsetzung:**

Nichtsyndromale orale Spaltbildungen (NSOS) resultieren aus Störungen in der primären und sekundären Gaumenentwicklung und werden in Lippenspalten (LS), Lippen-Kiefer-Gaumen-Spalten (LKGS) und Gaumenspalten (GS) eingeteilt. NSOS sind komplexe genetisch heterogene Erkrankungen, an denen mehrere Gene beteiligt sein können. Daher wurde in dieser Studie untersucht, ob funktionelle Einzelnukleotid-Polymorphismen (SNPs) in Genen, die potenziell den Vitamin-D-Spiegel und den Parathormon(PTH)-Spiegel beeinflussen, mit NSOS assoziiert sind.

**Materialien und Methoden:**

Ausgewählt wurden brasilianische NSOS-Patienten mit CL, CLP und CP sowie eine Vergleichsgruppe. Anhand von Epithelzellen der Mundschleimhaut wurde DNA gewonnen. Neun SNPs in 5 Genen *(VDR*, *CYP27B1*, *CYP24A1*, *SEC23A* und *PTH*) wurden ausgewählt. Die Genotypisierung wurde mittels Echtzeit-Polymerase-Kettenreaktion (PCR) durchgeführt. Der Zusammenhang zwischen NSOS und Vitamin-D-bezogenen Polymorphismen wurde mit dem χ^2^-Test auf einem Signifikanzniveau von *p* < 0,005 (nach Bonferroni-Korrektur) analysiert.

**Ergebnisse:**

Die Stichprobe umfasste 274 Personen: 154 NSOS-Patienten und 120 Kontrollpersonen. Insgesamt hatten 43 (27,9 %) CL, 91 (59,1 %) CLP und 20 (13,0 %) CP. Nach Bonferroni-Korrektur war keiner der SNPs mit NSOS assoziiert (*p* > 0,005).

**Schlussfolgerung:**

Keiner der untersuchten SNPs in den Genen, die mit Vitamin D und Parathormon in Verbindung stehen, war mit NSOS assoziiert.

## Introduction

Oral clefts are craniofacial congenital anomalies and are one of the most common congenital malformations in neonates worldwide, presenting in syndromic or nonsyndromic forms. Nonsyndromic oral clefts (NSOC) result from disruptions in the development of the primary and secondary palate [20]. NSOC are categorized into cleft lip (CL), cleft lip and palate (CLP) and cleft palate (CP). Since the lip and primary palate originate from a different embryological process than the secondary palate, CL and CLP are typically grouped as cleft lip with or without palate (CL ± P) [19]. NSOC is a condition with complex genetically heterogeneous backgrounds. Many studies have looked into the identification of genetic loci and candidate genes associated with NSOC adopting different approaches [3]. Although genetic variations in several candidate genes have been found, the exact etiology of NSOC still remains unknown [2].

Parathyroid hormone (PTH), calcium and vitamin D form a tightly regulated feedback loop essential for maintaining mineral homeostasis, where low blood calcium levels trigger PTH release that then stimulates active vitamin D synthesis. Both hormones work to raise calcium levels while active vitamin D conversely inhibits PTH secretion. Vitamin D, calcium and PTH play a crucial role in various physiological processes including craniofacial development. Engström et al. [29] explored the influence of a low calcium and vitamin D deficient diet on the growth and form of the craniofacial complex of growing rats. The authors observed that this diet also caused changes in cranial dimensions. The authors observed that the vitamin D free and low calcium diet caused disturbed osteogenesis in craniofacial growth sites. A study by Tsutsui et al. [30] with a transgenic mouse carrying the constitutively active PTH/PTHrP receptor observed abnormal formation of craniofacial bones.

Sunlight exposure and vitamin D levels has been hypothesized by Chung et al. as a reason for the seasonal variation in NSOC [6]. The concentration of vitamin D in the serum is primarily influenced by environmental and lifestyle factors such as sunlight exposure and genetic factors where gene – environment interactions may play a key role in vitamin D status [22]. A recent study investigated that serum levels of vitamin D in children with NSOC belonging to the Sub-Himalayan and Gangetic Plain regions were compared with age-matched noncleft children. The authors observed that vitamin D levels in NSOC children were significantly lower than in the control group [27]. This suggests that vitamin related genes could be involved in the etiology of NSOC. The current evidence supports that genetic factors such as single nucleotide polymorphisms (SNPs) play a significant role in determining vitamin D levels. A systematic review identified 35 genes that may modulate vitamin D serum concentrations and multiple SNPs within these genes have been linked to various clinical conditions [21]. Notable examples include the protein coding genes for vitamin D receptor (*VDR*), cytochrome P450 family 27 subfamily B member 1 (*CYP27B1*), cytochrome P450 family 24 subfamily A member 1 (CYP24A1), vitamin D binding protein (*VDBP*), SEC23 homolog A (*SEC23A*) and parathyroid hormone (*PTH*). Thus, the investigation of functional SNPs in these genes influencing the vitamin D [1, 11, 12, 18, 24, 25, 27] and PTH levels [8, 13, 23] and their association with NSOC was the aim of the present study.

## Materials and methods

### Study design and ethical aspects

This manuscript features a cross-sectional study design. It is a part of a larger project that was reported according to the Strengthening the Reporting of Genetic Association Study Statement (STREGA) checklist [17]. The study protocol followed the principles of the Declaration of Helsinki and was approved by the Research Ethics Committee of the State Health Department of Paraná (protocol number 5.100.185) and by the Human Research Ethics Committee of the Health Sciences of the Federal University of Paraná (protocol number 3.752.172). Assent was obtained from the children to ensure their understanding and willingness to participate while informed consent was secured from their legal guardians, granting official permission for their involvement in the study.

The sample size calculation was based on the frequencies of genotypes for that a minimum of 232 (116 cases and 116 controls) individuals were calculated to be required (frequency difference = 0.17, degrees of freedom = 1, α = 0.05 and β = 0.80).

### NSOC definition

Individuals with NSOC were recruited at the “Comprehensive Care Center for Cleft Lip and Palate” in Curitiba, Paraná, Brazil, while they were undergoing treatment at this specialized center for craniofacial anomalies. The comparison group of individuals without NSOC was recruited in the pediatric dentistry clinic at the Federal University of Paraná that is also located in Curitiba, Paraná, Brazil. Individuals in both groups were selected through convenience sampling. Children with syndromes or craniofacial anomalies other than oral clefts and dental anomalies were not included in this study.

The sample recruitment occurred between November 2020 and August 2023. The study included only biologically unrelated individuals (one patient per family). The age of the included individuals ranged from 5 to 14 years old and all included patients presented repaired clefts. Individuals with syndromes or craniofacial anomalies other than oral clefts were excluded.

The diagnosis of NSOC (cleft lip, cleft lip with palate, cleft palate) was primarily determined by the pediatrician through visual inspection and physical examination of the infant’s face and oral cavity before surgery for identifying visible separations in the lip and/or palate structures. This information regarding the type of cleft was noted in the child’s medical record. A dentist also performed a clinical examination during the day of the saliva collection. The examination during the saliva collection was done with visual inspection and physical examination of the child’s face and oral cavity after cleft and/or palate primary repair. The diagnosis in the medical record was 100% in agreement with the dentist diagnosis. Oral clefts were classified into three groups: cleft lip only (CL), cleft palate only (CP) and cleft lip and palate (CLP) [16].

### DNA isolation and genotyping

A 5 ml saline mouthwash was used to collect the cells. The genomic DNA was isolated from these cells following the protocol outlined by Küchler et al. in 2012 [15]. DNA concentration and purity were assessed via spectrophotometry using Nanodrop 2000 (Thermo Scientific, Wilmington, DE, USA). Genotyping was performed through real-time polymerase chain reaction (RT-PCR) using the StepOnePlus Real-Time PCR System (Thermo Fisher Scientific, Waltham, MA, USA). The reaction mixture consisted of 3.1 µL total volume, including 1.5 µL of diluted DNA sample (5 ng/µL), 1.5 µL of TaqMan Genotyping Master Mix and 0.095 µL of predesigned TaqMan probes and primers (all reagents are from Applied Biosystems, Foster City, CA, USA). The thermal cycling process included two stages: a hold stage at 95 °C for 10 min, followed by amplification cycling at 92 °C for 15 s and 60 °C for 60 s as reported in Fonseca-Souza et al. [9]. A negative control template was included in each reaction plate. In addition, 10% of the samples were randomly selected for repeated analysis and the results showed 100% concordance. DNA samples that could not be genotyped were excluded from further analyses.

SNPs were selected due to their reported function in influencing vitamin D and PTH levels. Potentially functional SNPs were screened from the dbSNP database (http://www.ncbi.nlm.nih.gov/snp/) and SNPinfo (http://snpinfo.niehs.nih.gov/). The characteristics of the selected SNPs are presented in Table 1.

**Table 1:** Characteristics of the studied single nucleotide polymorphisms (SNPs) Merkmale der untersuchten Einzelnukleotid-Polymorphismen (SNPs)

Chromosome	Acronym	Gene	SNP	Base change	Reported effects
12q13.11	*VDR*	*Vitamin D receptor*	rs1544410	C**T** (Bsml)	Involved in high serum levels of vitamin D [18]
			rs7975232	A**C** (Apal)	Involved in high serum levels of vitamin D [1]
			rs2228570	**A**G (Fokl)	Involved in low serum levels of vitamin D [24]
12q14.1	*CYP27B1*	*Cytochrome P450 family 27 subfamily B member 1*	rs4646536	A**G**	Involved in high serum levels of vitamin D [25]
20q13.2	*CYP24A1*	*Cytochrome P450 family 24 subfamily A member 1*	rs927650	C**T**	Involved in high serum levels of vitamin D [11]
14q21.1	*SEC23A*	*SEC23 homolog A*	rs8018720	C**G**	Involved in low serum levels of vitamin D [12]
11p15.3	*PTH*	*Parathyroid hormone*	rs307247	**A**G	Involved in low serum levels of PTH [23]
			rs694	**C**T	Involved in low serum levels of PTH [8]
			rs6256	**G**T	Involved in low serum levels of PTH [13]

Note: Bold letter indicates minor allele

### Statistical analysis

Chi-square (Χ^2^) calculations were used to assess Hardy–Weinberg equilibrium (HWE) for each SNP (wpcalc.com/en/equilibrium-hardy-weinberg). Deviations from HWE were considered when Χ^2^ was higher than 3.84.

In order to evaluate the association between genotype frequencies for each SNP in the codominant model and also in the recessive and dominant models, Χ^2^ or Fisher’s exact tests were used. The recessive and dominant genetic models are applied by grouping genotypes based on assumed allele effects. The recessive model groups homozygotes for the risk allele separately; the dominant model groups genotypes with at least one risk allele. Data were analyzed using Epi Info 3.5.2 (Atlanta, GA, USA). (www.cdc.gov/epiinfo). To account for multiple testing, the Bonferroni correction was applied (0.05/9 SNPs = 0.0055). Consequently, statistical significance was established at an α level of ≤ 0.005.

## Results

The final sample consisted of 274 individuals, 154 (88 males and 66 females) NSOC individuals and 120 (53 males and 67 females) controls (non-NSOC). A total of 43 (27.9%) had CL, 91 (59.1%) had CLP and 20 (13.0%) had CP.

The genotype data were in Hardy–Weinberg equilibrium (HWE) for all studied SNPs in the control and NSOC groups (Χ^2^ HWE < 3.84).

Table 2 shows the genotype distribution according to the groups. A borderline result was observed for the rs694 for NSOC compared to the control (*p* = 0.0243) and for the CL compared to the control (*p* = 0.0302) and for CL + CLP compared to the control (*p* = 0.048). The CC genotype was more frequent in the control group. After Bonferroni correction, none of the SNPs were associated with NSOC (*p* > 0.005).

**Table 2:** Genotype distribution according to the groups Genotyp-Verteilung in den einzelnen Gruppen

Genes	SNPs	Control *N* (%)			NSOC (%)			CL *N* (%)			CLP *N* (%)			CL + CLP *N* (%)			CP *N* (%)		
***VDR***	**rs1544410**	**TT**	**TC**	**CC**	**TT**	**TC**	**CC**	**TT**	**TC**	**CC**	**TT**	**TC**	**CC**	**TT**	**TC**	**CC**	**TT**	**TC**	**CC**
		16 (13.8)	48 (41.4)	52 (44.8)	18 (12.7)	70 (49.3)	54 (38.0)	9 (22.5)	18 (45.0)	13 (32.5)	5 (6.0)	45 (53.6)	34 (40.5)	14 (11.3)	63 (50.8)	47 (37.9)	4 (22.2)	7 (38.9)	7 (38.9)
	***p‑values***	Reference			*p* = 0.437			*p* = 0.275			*p* = 0.0990			*p* = 0.341			*p* = 0.641		
	**rs7975232**	**AA**	**AC**	**CC**	**AA**	**AC**	**CC**	**AA**	**AC**	**CC**	**AA**	**AC**	**CC**	**AA**	**AC**	**CC**	**AA**	**AC**	**CC**
		43 (37.7)	46 (40.4)	25 (21.9)	51 (36.7)	64 (46.0)	24 (17.3)	17 (43.6)	17 (43.6)	5 (12.8)	27 (32.5)	40 (48.2)	16 (19.3)	44 (36.1)	57 (46.7)	21 (17.2)	7 (41.2)	7 (41.2)	3 (17.7)
	***p‑values***	Reference			*p* = 0.552			*p* = 0.459			*p* = 0.548			*p* = 0.534			*p* = 0.916		
	**rs2228570**	**AA**	**AG**	**GG**	**AA**	**AG**	**GG**	**AA**	**AG**	**GG**	**AA**	**AG**	**GG**	**AA**	**AG**	**GG**	**AA**	**AG**	**GG**
		14 (12.2)	58 (50.4)	43 (37.4)	21 (14.8)	67 (47.2)	54 (38.0)	5 (12.2)	19 (46.3)	17 (41.5)	13 (15.7)	36 (43.4)	34 (41.0)	18 (14.5)	55 (44.4)	51 (41.1)	3 (16.7)	12 (66.7)	3 (16.7)
	***p‑value***	Reference			*p* = 0.793			*p* = 0.890			*p* = 0.578			*p* = 0.603			*p* = 0.228		
***CYP27B1***	**rs4646536**	**AA**	**AG**	**GG**	**AA**	**AG**	**GG**	**AA**	**AG**	**GG**	**AA**	**AG**	**GG**	**AA**	**AG**	**GG**	**AA**	**AG**	**GG**
		61 (55.0)	40 (36.0)	10 (9.0)	77 (55.8)	49 (35.5)	12 (8.7)	21 (53.9)	14 (35.9)	4 (10.3)	46 (56.1)	29 (35.4)	7 (8.5)	67 (55.4)	43 (35.5)	11 (9.1)	10 (58.8)	6 (35.3)	1 (5.9)
	***p‑values***	Reference			*p* = 0.990			*p* = 0.973			*p* = 0.986			*p* = 0.997			*p* = 0.900		
***CYP24A1***	**rs927650**	**CC**	**CT**	**TT**	**CC**	**CT**	**TT**	**CC**	**CT**	**TT**	**CC**	**CT**	**TT**	**CC**	**CT**	**TT**	**CC**	**CT**	**TT**
		28 (24.4)	61 (53.0)	26 (22.6)	41 (28.7)	67 (46.9)	35 (24.5)	12 (29.3)	16 (39.0)	13 (31.7)	26 (31.0)	38 (45.2)	20 (23.8)	38 (30.4)	54 (43.2)	33 (26.4)	3 (16.7)	13 (72.2)	2 (11.1)
	***p‑values***	Reference			*p* = 0.597			*p* = 0.288			*p* = 0.495			*p* = 0.307			*p* = 0.301		
***SEC23A***	**rs8018720**	**CC**	**CG**	**GG**	**CC**	**CG**	**GG**	**CC**	**CG**	**GG**	**CC**	**CG**	**GG**	**CC**	**CG**	**GG**	**CC**	**CG**	**GG**
		84 (73.0)	28 (24.4)	3 (2.6)	95 (66.0)	43 (29.9)	6 (4.2)	26 (63.4)	11 (26.8)	4 (9.8)	55 (64.7)	28 (32.9)	2 (2.4)	81 (59.6)	49 (36.0)	6 (4.4)	14 (77.8)	4 (22.2)	0 (0.0)
	***p‑values***	Reference			*p* = 0.445			*p* = 0.141			*p* = 0.408			*p* = 0.079			*p* = 0.761		
***PTH***	**rs307247**	**AA**	**AG**	**GG**	**AA**	**AG**	**GG**	**AA**	**AG**	**GG**	**AA**	**AG**	**GG**	**AA**	**AG**	**GG**	**AA**	**AG**	**GG**
		22 (19.1)	55 (47.8)	38 (33.0)	19 (13.2)	61 (42.4)	64 (44.4)	5 (12.2)	17 (41.5)	19 (46.3)	13 (15.3)	36 (42.4)	36 (42.4)	18 (14.3)	53 (42.1)	55 (43.7)	1 (5.6)	8 (44.4)	9 (50.0)
	***p‑values***	Reference			*p* = 0.138			*p* = 0.277			*p* = 0.391			*p* = 0.217			*p* = 0.227		
	**rs694**	**CC**	**CT**	**TT**	**CC**	**CT**	**TT**	**CC**	**CT**	**TT**	**CC**	**CT**	**TT**	**CC**	**CT**	**TT**	**CC**	**CT**	**TT**
		31 (26.7)	57 (49.1)	28 (24.1)	21 (14.6)	72 (50.0)	51 (35.4)	5 (12.2)	18 (43.9)	18 (43.9)	14 (16.5)	46 (54.1)	25 (29.4)	19 (15.1)	64 (50.8)	43 (34.1)	2 (11.1)	8 (44.4)	8 (44.4)
	***p‑values***	Reference			*p* = 0.0243			*p* = 0.0302			*p* = 0.217			*p* = 0.048			*p* = 0.135		
	**rs6256**	**GG**	**GT**	**TT**	**GG**	**GT**	**TT**	**GG**	**GT**	**TT**	**GG**	**GT**	**TT**	**GG**	**GT**	**TT**	**GG**	**GT**	**TT**
		84 (73.7)	25 (21.9)	5 (4.4)	110 (76.9)	30 (21.0)	3 (2.1)	35 (85.4)	6 (14.6)	0 (0.0)	62 (73.8)	20 (23.8)	2 (2.4)	107 (79.3)	26 (19.3)	2 (1.5)	13 (72.2)	4 (22.2)	1 (5.6)
	***p‑values***	Reference			*p* = 0.554			*p* = 0.208			*p* = 0.732			*p* = 0.313			*p* = 0.974		

Note: All comparisons were performed with the control (reference)

*SNPs* single nucleotide polymorphisms

## Discussion

NSOC is considered one of the most common birth defects worldwide. NSOC including CL, CP and CLP affect multiple craniofacial structures leading to significant physiological difficulties that affect feeding, speech and language development that results in an increased social and financial burden on the affected individuals and their families. The management of NSOC is based on a multidisciplinary team approach and orthodontists play a crucial role in the treatment of oral clefts. Since clefts affect the development of the maxilla, teeth and occlusion, orthodontic intervention is essential at multiple stages of treatment as part of a multidisciplinary cleft care team. Therefore, we decided to investigate vitamin D related genes in the current study to better understand the genetic factors influencing NSOC and to identify potential risk variants that contribute to the occurrence of this condition.

We investigated 9 SNPs within 5 genes that have been previously suggested to be associated with the regulation of vitamin D levels [1, 11, 12, 18, 24, 25, 27] and PTH levels [8, 13, 23]. The candidate gene approach aids to identify risk variants/SNPs. The selection of the candidate genes to study depends on having previous knowledge about the molecular pathways and interaction networks involved in the cleft formation by selecting the genes known to be involved or expressed in facial development. It is important to emphasize that candidate gene studies are targeted genetic analyses that depend on isolated cases of affected individuals. This approach searches for a statistically significant association between a genotype and the specific phenotype (NSOC) and subphenotypes (CL, CLP and CP) and the genotype frequency variation between affected and unaffected individuals. Although samples of the control group could not be collected in the same medical center as for those of the individuals with NSOC, the control was collected in a dental clinic located in the same city in order to match the two groups as much as possible.

One important aspect is that around 70% of the cases of cleft lip and/or cleft palate are of the nonsyndromic type [3]. In our study, only nonsyndromic cases were included because of syndromic clefts are often caused by single gene mutations or chromosomal abnormalities that are often already characterized well. Therefore, in the current project we focused on NSOC that had a diverse genetic background influenced by several gene – gene and gene – environment interactions [3]. In our study, none of the SNPs were associated with NSOC after Bonferroni correction. The generalizability of our data is one important aspect to be discussed. An association between an SNP and NSOC identified in one population often cannot be directly generalized to other populations due to several key factors. Genetic heterogeneity means that the underlying genetic causes of clefting can differ significantly between distinct ancestral groups. An SNP that acts as a strong risk factor in one population might have a weaker or no effect in another one because different genes or combinations of genes contribute to the condition’s development in different ethnic backgrounds. Differences in allele frequencies and linkage disequilibrium patterns across populations play a crucial role. Additionally, gene – environment interactions can also contribute to this lack of generalizability. The influence of a SNP on cleft risk might be modulated by environmental factors that differ in prevalence or type across populations [19].

A connection between clefting and PTH was observed in a genetic condition: the DiGeorge syndrome (22q11.2 deletion syndrome). This condition commonly leads to several developmental problems including disorders of PTH metabolism and cleft palate [28]. The rs694, an SNP in a gene encoding parathyroid hormone, was borderline associated with NSOC for that a significance was observed only before Bonferroni correction. PTH is important for craniofacial development. SNPs in *PTH* were previously associated with craniofacial morphology [14] and dental morphology [10]. In our study, the CC genotype presented *p*-values less than 0.05 but greater than 0.0055. Therefore, a statistical association was not reported. We must emphasize that the observed result may represent a false negative (type II error), a consequence of employing a post hoc test designed to control the family-wise error rate that was required due to multiple testing. This means we might have incorrectly described a lack of association. Post hoc tests such as the Bonferroni correction used for this study are simple and effective at preventing false positives across multiple tests but they are often criticized for being overly cautious. Its main limitations include significantly reducing statistical power (increasing false negatives) by setting a very strict threshold for significance, especially with many tests as performed in the current study. This can lead to missing real findings and is generally not recommended for exploratory research.

The studied SNPs in the vitamin related genes were not associated with NSOC. The biological actions of vitamin D are employed by binding its nuclear receptor (VDR) [7] that is expressed in the parathyroid glands acting as sensors for the detection and maintenance of vitamin D levels, adjusting PTH release and synthesis [4]. Moreover, additional molecules such as the enzymes *CYP24A1, CYP27B1, VDBP* and *SEC23A* are pivotal determinants of the local concentration of vitamin D [26]. The lack of association observed in the current study could be explained by the genetic heterogeneity of NSOC, the contribution of environmental factors and gene – environment interaction. Another important aspect to be highlighted here is the SNPs selection. Since this is a preliminary study and there is no previous evidence of their association with NSOC, the selection of the SNPs was based on their functional role on vitamin D and PTH levels [1, 8, 11–13, 18, 23–25].

Briefly, craniofacial development is a highly complicated process involving a combination of complex interactions between molecular signals and transcription factors that patterns the morphogenesis [5]. Disruption in early embryogenesis could result in the failure of the proper fusion of the facial prominences resulting in NSOC. Continued genetic research on NSOC is essential for improving prevention, diagnosis and treatment.

## Conclusion

The studied single nucleotide polymorphisms (SNPs) of the genes encoding parathyroid hormone (PTH) and vitamin D related genes were not associated with nonsyndromic oral cleft (NSOC) in Brazilian children.

## Data Availability

The data that support the findings of this study are not openly available due to reasons of sensitivity and are available from the corresponding author upon reasonable request.
